# Diagnostic and prognostic potential of miR-21, miR-29c, miR-148 and miR-203 in adenocarcinoma and squamous cell carcinoma of esophagus

**DOI:** 10.1186/s13000-015-0280-6

**Published:** 2015-04-28

**Authors:** Renata Hezova, Alena Kovarikova, Josef Srovnal, Milada Zemanova, Tomas Harustiak, Jiri Ehrmann, Marian Hajduch, Marek Svoboda, Milana Sachlova, Ondrej Slaby

**Affiliations:** Molecular Oncology II – Solid Cancers, Molecular Medicine Central European Institute of Technology, Masaryk University, Brno, Czech Republic; Department of Comprehensive Cancer Care, Masaryk Memorial Cancer Institute, Zluty kopec 7, Brno, 656 53 Czech Republic; Institute of Molecular and Translational Medicine, Faculty of Medicine and Dentistry, Palacky University Olomouc, Olomouc, 779 00 Czech Republic; Department of Oncology, General University Hospital in Prague, Prague, Czech Republic; 3rd Department of Surgery, University Hospital in Motol, Prague, Czech Republic; Department of Histology and Embryology, Faculty of Medicine and Dentistry, Palacky University and University Hospital, Olomouc, 779 00 Czech Republic

## Abstract

**Background:**

Esophageal cancer is the malignant tumor with very poor prognosis and increasing incidence often diagnosed at very late stage, so the prognosis of affected patients is unsatisfactory, despite the development of therapeutic option such as surgery, chemotherapy and radiotherapy. Consequently, there is a great need for biomarkers to allow a tailored multimodality approach with increased efficiency. Altered expression of microRNAs has been reported in wide range of malignancies, including esophageal cancer. The aim of this study was to examine the expression levels of candidate microRNAs in esophageal cancer and evaluate their diagnostic and prognostic potential.

**Findings:**

Using quantitative real-time PCR, expression levels of 9 candidate microRNAs were examined in 62 tissue samples, 23 esophageal adenocarcinomas, 22 esophageal squamous cell carcinomas and 17 adjacent esophageal mucosa samples. MicroRNA expression levels were further analyzed in regards to clinico-pathological features of esophageal cancer patients. We observed significantly decreased levels of miR-203 and increased levels of miR-21 in adenocarcinoma tissues when compared to normal mucosa. MiR-29c and miR-148 indicated good ability to distinguish between histological subtypes of esophageal cancer. MiR-203 and miR-148 were linked to disease-free survival and overall survival in esophageal adenocarcinoma patients, and miR-148 also in esophageal squamous cell carcinoma patients.

**Conclusions:**

Our data suggest that altered expression of miR-21, miR-29c, miR-148 and miR-203 are related to neoplastic transformation and progression of the disease and these microRNAs could serve as a potential diagnostic and prognostic biomarkers in esophageal cancer.

**Virtual slides:**

The virtual slide(s) for this article can be found here: http://www.diagnosticpathology.diagnomx.eu/vs/4646922201567057

## Findings

### Background

Esophageal cancer (EC) is the seventh most common cancer worldwide and the sixth most common cause of cancer death [1]. There are two main types of esophageal cancer – adenocarcinoma and squamous cell carcinoma with distinct etiology and epidemiology.

Esophageal adenocarcinoma (EAC) is one of the fastest rising cancers in Western society. Incidence has increased by 600% within the last 30 years [2]. The reason of increasing incidence is not entirely clear but the main risk factors are male sex (5 more often than female sex), Caucasian race, chronic reflux disease and visceral obesity [2]. In contrast, esophageal squamous cell carcinoma (ESCC) is more prevalent in the developing world with very high incidence areas found in East Asia mainly in China [3]. While the main risk factors are smoking and alcohol consumption, high diets in N-nitroso compounds, fungal toxins, low in selenium, zinc, vitamins A and C and highly salted meats are also discussed as potential risk factors.

Both EAC and ESCC are usually detected at an advanced stage, requiring a multimodal concept of therapy, however the overall survival of esophageal cancer remains lower than other solid tumors [4]. Although the treatments have made great progress, the prognosis for patients with advanced disease still remains poor and unsatisfactory [5]. Potentially curative treatments are followed by high rates of disease recurrence. All these facts together clearly indicate the need for a molecular biomarker for esophageal cancer enabling earlier detection and prognostic stratification.

MicroRNA (miRNA) are short non-coding RNA with ability to regulate important cellular processes as differentiation, cell cycle, proliferation or apoptosis. They are known to be deregulated in most tumor types [6], in which they can act as tumor suppressors or oncogenes. Altered miRNA expression profiles are intensively studied also in esophageal cancer. In this study, we hypothesized that selected miRNAs can be identified as diagnostic and/or prognostic biomarkers in EC. Thus, we analyzed 9 candidate miRNAs in EAC, ESCC and non-tumor esophageal mucosa and investigated their potential for diagnostic and prognostic usage in EC.

### Methods

#### Study population

A total of 62 tissue samples from two esophageal cancer cohorts were included in the study. First cohort (Motol University Hospital, 3rd Department of Surgery, Prague, Czech Republic) consisted of 17 EAC and 5 ESCC cases, whereas for 17 EAC cases paired esophageal mucosa samples were available. Second cohort (Palacky University, Medical Faculty, Olomouc, Czech Republic) consisted of 6 EAC and 17 ESCC cases. All subjects were of the same ethnicity (Caucasian). Clinico-pathological features including age, sex, histology and treatment were recorded and summarized in Table 1. Written informed consent was obtained from the patients before starting this study. The study has been approved by the local ethical committees (Prague, Olomouc).

**Table 1:** **Patient characteristics**

**Characteristic**			**Prague cohort**	**Olomouc cohort**
**Total**			**22**	**23**
**Gender**	Male		**20**	**18**
	Female		**2**	**5**
**Age**			**59 (49–70)**	**58 (34–79)**
**Histology**	Adenocarcinoma		**17**	**6**
	Squamous cell carcinoma		**5**	**17**
**Pre-operative chemotherapy**	Adenocarcinoma	**CDDP+FU 2x**	**0**	**6**
		**ECF/ECX**	**17**	**0**
	Squamous Cell carcinoma	**CDDP+FU 2x**	**3**	**17**
		**ECF/ECX**	**2**	**0**
**Tumor location**	Upper esophagus		**0**	**0**
	Middle esophagus		**20**	**11**
	Lower esophagus		**2**	**12**

#### Tissue sample preparation and miRNA isolation

Forty-five samples of tumor tissue and seventeen samples of non-cancerous esophageal mucosa were collected and stored in RNAlater (Ambion, USA). Afterward, tissue samples were homogenized (MagnaLyser, Roche), and total RNA enriched for small RNA fraction was isolated using fenol-chloroform extraction with TRIzol reagent (Ambion, USA). Concentration and purity of RNA were determined using a Nanodrop ND-1000 (Thermo Scientific, USA), and RNA integrity was measured by Agilent 2100 Bioanalyzer using Agilent RNA 6000 Nano Kit (Agilent Technologies, USA). Samples were either stored at -80°C or further processed.

#### Quantitative real-time PCR

cDNA was synthesized from total RNA using miRNA-specific primers according to the Taq-Man MicroRNA Assay protocol (Applied Biosystems, USA). Real-time polymerase chain reaction (PCR) was performed according to manufacturing protocol (MicroRNA Assay, Applied Biosystems, USA) using the Applied Biosystems 7500 Instrument as described previously [7]. All reactions were run in triplicate, and average threshold cycle and SD values were calculated.

#### Data normalization and statistical analysis

The threshold cycle data were calculated by SDS 2.0.1 software (Applied Biosystems, USA). The average expression levels of measured miRNAs were normalized using RNU44 as reference gene and subsequently analyzed by the 2^-ΔCt^ method. Differences between expression levels in paired samples were evaluated by non-parametric Wilcoxon test. Differences between miRNA levels in EAC and ESCC samples were evaluated by non-parametric Mann–Whitney *U*-test. Survival analyses were carried out using the log-rank test and Kaplan-Meier survival analysis. All calculations were performed using Prism version 6.00 (GraphPad Software, San Diego, CA, USA). P-values of less than 0.05 were considered statistically significant.

### Results

#### miR-203 and miR-21 are able to distinguish EAC and normal tissue

**Figure 1:**
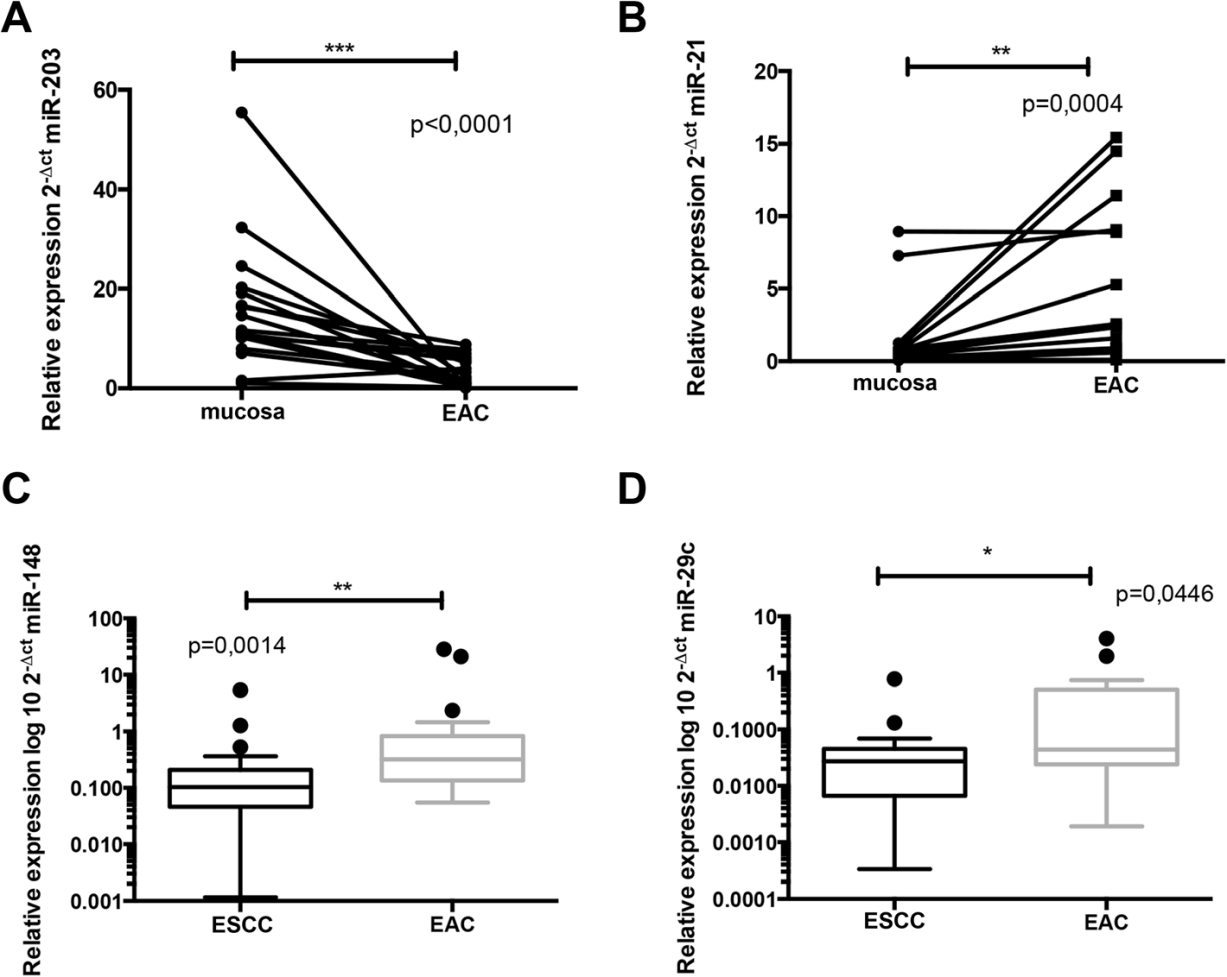
Normalized expression levels of miR-203 and miR-21 in esophageal adenocarcinoma tissue and control mucosa. **A)** MiR-203 is decreased in esophageal adenocarcinoma compared to paired adjacent mucosa (P < 0.0001). **B)** MiR-21 is increased in esophageal adenocarcinoma compared to paired adjacent mucosa (P = 0.0004). **C)** MiR-148a is up-regulated in esophageal adenocarcinoma compared to esophageal squamous cell carcinoma (P = 0.0014). **D)** MiR-29c is up-regulated in esophageal adenocarcinoma compared to esophageal squamous cell carcinoma (P = 0.0446). EAC – esophageal adenocarcinoma; ESCC – esophagus squamous cell carcinoma; *P < 0.05; **P < 0.001; ***P < 0.0001

To determine whether the selected miRNAs (miR-200c, miR-25, miR-27a, miR-7a, miR-29c, miR-203, miR-148a, miR-21 a miR-31) have a diagnostic potential, expression levels were analyzed for each miRNA in 17 EAC samples and 17 paired adjacent mucosa. There were 2 miRNAs differentially expressed in tumor tissue and adjacent mucosa. MiR-203 levels were significantly down-regulated in tumor tissue (fold change 0,19; p < 0,0001), miR-21 levels were significantly up-regulated in tumor tissue (fold change 40,38; p = 0,0004) (Figure 1 A,B). No significant difference was observed in the other examined miRNA as summarized in Table 2.

**Table 2:** **MiRNA expression levels in adenocarcinoma and paired non-tumoral esophageal mucosa**

	**Adenocarcinoma (n = 17)**	**Mucosa (n = 17)**	**FC**	**p-value**
	**Median**	**Median**		
	**(25%–75% percentile)**	**(25%–75% percentile)**		
**miR-203**	2,312	12,28	0,19	**< 0,0001**
	(0,5304–5,990)	(9,139–19,73)		
**miR-21**	2,463	0,06064	40,38	**0,0004**
	(0,8149–9,648)	(0,06729–0,9838)		
miR-25	0,07557	0,07959	0,95	0,6221
	(0,02829–0,3545)	(0,02275–0,2205)		
miR-27a	1,763	2,74	0,64	0,1006
	(1,236–3,482)	(2,096–5,082)		
miR-31	0,6739	0,4659	1,45	0,1201
	(0,3100–2,596)	(0,4084–0,8982)		
miR-200c	1,718	1,884	0,92	0,2036
	(1,182–2,705)	(1,496–4,017)		
let-7a	0,15	0,196	0,77	0,3394
	(0,1143–0,4092)	(0,04641–0,2439)		
miR-29c	0,03389	0,1647	0,21	0,9697
	(0,01787–0,7736)	(0,0233–0,6866)		
miR-148	0,1779	0,1785	1,00	0,1893
	(0,1244–0,3107)	(0,1050–0,5636)		

MiRNAs with significant differences are bolded.

#### miR-148 and miR-29c have ability to distinguish EAC and ESCC tissues

To determine whether different expression levels of analyzed miRNAs exist in EAC and ESCC tissues, we performed qPCR analysis of selected miRNAs (miR-200c, miR-25, miR-27a, miR-7a, miR-29c, miR-203, miR-148a, miR-21 a miR-31) in 22 ESCC samples and 22 EAC samples. We found that miR-148 and miR-29c were decreased in ESCC when compared to EAC and have ability to significantly distinguish ESCC and EAC tissues (fold change 0,32; p = 0,0014; fold change 0,62; p = 0,0446), respectively (Figure 1C,D). No significant difference was observed in the other examined miRNA as summarized in Table 3.

**Table 3:** **Comparison of miRNAs expression levels in ESCC and EAC tissues**

	**Esophageal squamous cell carcinoma (n = 22)**	**Adenocarcinoma (n = 22)**	**FC**	**p-value**
	**Median**	**Median**		
	**(25%–75% percentile)**	**(25%–75% percentile)**		
**miR-148**	0,1031	0,3213	0,32	**0,0014**
	(0,04630–0,2080)	(0,1358–0,8261)		
**miR-29c**	0,02725	0,04402	0,62	**0,0446**
	(0,006621–0,04527)	(0,02413–0,5075)		
miR-21	0,8619	0,9001	0,96	0,2093
	(0,3963–2,328)	(0,6343–6,177)		
miR-25	0,222	0,1495	1,48	0,9266
	(0,009942–0,5544)	(0,03441–0,4671)		
miR-27a	3,802	1,592	2,39	0,1333
	(1,563–7,393)	(1,168–3,997)		
miR-31	0,8068	0,5517	1,46	0,8971
	(0,3731–1,394)	(0,31–2,596)		
miR-200c	1,681	2,155	0,78	0,6304
	(0,8033–3,385)	(1,201–3,419)		
let-7a	0,4739	0,1588	2,98	0,1168
	(0,1811–0,9559)	(0,1177–0,3838)		
miR-203	3,976	2,1	1,89	0,6304
	(0,3679–14,51)	(0,1074–5,347)		

MiRNAs with significant differences are bolded.

#### MiR-148 and miR-203 are associated with prognosis in EC patients

**Figure 2:**
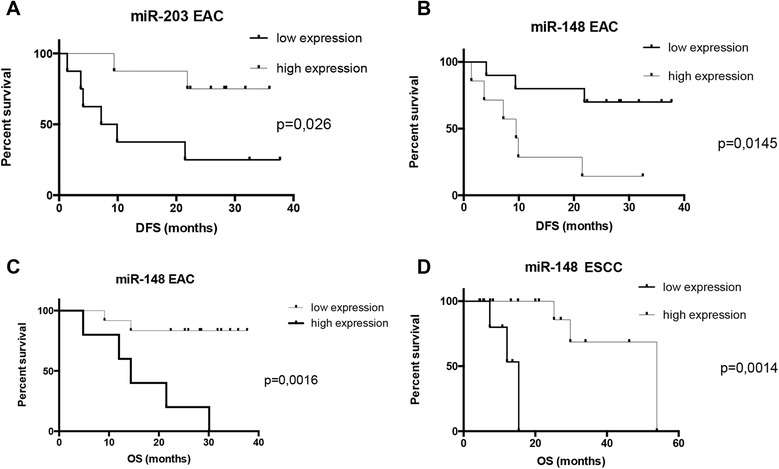
Survival analysis of miR-203 and miR-148 tissue levels in esophageal squamous cell carcinoma and esophageal adenocarcinoma patients. **A)** Esophageal adenocarcinoma patients with low level of miR-203 have shorter disease free survival (P = 0.026). **B)** Esophageal adenocarcinoma patients with high level of miR-148 have shorter disease free survival (P = 0.0145). **C)** Esophageal adenocarcinoma patients with high level of miR-148 have shorter overall survival (P = 0.0016). **D)** Esophageal squamous cell carcinoma patients with low level of miR-148 have shorter overall survival (P = 0.0014). DFS – disease free survival; OS – overall survival.

EAC patients with decreased miR-203 levels showed significantly shorter disease free survival (p = 0,026). Down-regulation of miR-148 was associated with significantly shorter overall survival (p = 0,0014) in ESCC patients. Interestingly, opposite observation was reached in EAC patients, where cases with up-regulation of miR-148 showed significantly shorter disease free survival (p = 0,0145) and overall survival (p = 0,0016), indicating opposite functioning of miR-148 in EAC and ESCC (Figure 2A-D). There was no association with survival observed in other examined miRNAs.

### Discussion

Our results indicate that miR-203 plays role of tumor suppressor in EAC since the expression levels of this miRNA were significantly decreased in tumor tissue of EAC patients. Those results are in compliance with another studies, where miR-203 displayed stepwise decrease in expression during malignant progression from healthy tissue through Barrett’s esophagus to EAC [8-11]. Moreover, survival analysis showed significantly shorter disease free survival of the EAC patients with low expression of miR-203 in our study. Another miRNA observed to have different expression in EAC tissue and adjacent mucosa is miR-21, which is well known as oncogene. In our study, expression levels of miR-21 were significantly higher in EAC tissue, which is in concordance with the several previous studies [8,10-12]. We have not found association of miR-21 and survival in our study, which is contradictory to meta-analysis performed by Fu *et al.*, where up-regulation of miR-21 could predict unfavorable prognosis in esophageal cancer [12].

The second aim of our study was to discriminate EAC from ESCC tissue. For the first time, we found that the expression levels of miR-148a and miR-29c are significantly higher in EAC when compared to ESCC tissue. Moreover up-regulation of miR-148 could predict unfavorable prognosis in EAC cancer and surprisingly favorable prognosis in ESCC patients. It seems that miR-148 can play the role of tumor suppressor or tumor oncogene depending on histological subtype of esophageal cancer.

MiR-148 was not yet studied in association with esophageal cancer, but it is known to act as oncogene and also as tumor suppressor in different tumor types. Thus, Kim *et al.* has reported the definition of miR-148a as a novel prognostic oncomiR in glioblastoma with high levels considered as a risk factor for glioblastoma patients survival [13]. When analyzed in vitro, miR-148a overexpression increased cell growth, survival, migration, and invasion in glioblastoma cells [13]. On the other hand, in gastric cancer (GC), miR-148 acts as tumor suppressor and its down-regulation is linked to the poor prognosis. MiR-148a levels were found to be decreased in GC cell lines and in GC tissue samples when compared to the adjacent normal gastric tissues and in different clinical stages of GC [14-18]. Low expression of miR-148a was significantly correlated with an advanced clinical stages, lymph node metastasis, and poor clinical outcome [14].

Our data indicate, that miR-203 and miR-21 could serve as diagnostic biomarkers. Further, miR-29c and miR-148 have ability to distinguish between EAC and ESCC histological subtypes of esophageal cancer. In future, the diagnosis could be assessed for instance from non-invasive brush biopsy of esophagus [19]. Moreover miR-203 and miR-148 could serve as prognostic biomarker in EAC, and miR-148 also in ESCC patients. Further studies are needed to evaluate mechanistically dual functioning of miR-148 in EAC and ESCC carcinogenesis.

## Abbreviations

EAC: Esophageal adenocarcinoma
ESCC: Esophageal squamous cell carcinoma
MiRNA: MicroRNA
DFS: Disease free survival
OS: Overall survival
GC: Gastric cancer
